# Unravelling the Complexity of Myelin Oligodendrocyte Glycoprotein Antibody-Associated Disease

**DOI:** 10.7759/cureus.59840

**Published:** 2024-05-07

**Authors:** Husain Abbas, Prakhar Kumar, Abdullah, Razi Quamar, Utsav Anand Mani

**Affiliations:** 1 Internal Medicine, Jawaharlal Nehru Medical College and Hospital, Aligarh Muslim University, Aligarh, IND; 2 Emergency Medicine, Sanjay Gandhi Postgraduate Institute of Medical Sciences, Lucknow, IND

**Keywords:** azathioprine treatment, immunomodulator therapy, neuromyelitis optica spectrum disorder (nmosd), acute encephalitis, optic neuritis, acute disseminated encephalomyelitis, myelin oligodendrocyte glycoprotein, autoantibodies, demyelinating disorders, mogad

## Abstract

Myelin oligodendrocyte glycoprotein antibody-associated disease (MOGAD) is a rare autoimmune disorder characterized by recurrent episodes of demyelination affecting the central nervous system. The following case report showcases a thorough analysis of a 21-year-old female patient presenting with MOGAD, outlining her clinical presentation, diagnostic workup, treatment protocol, and long-term management outcomes. Through a multidisciplinary approach, we aim to augment the understanding of this complex neurological entity and steer optimal therapeutic interventions.

## Introduction

MOGAD, which stands for myelin oligodendrocyte glycoprotein (MOG) antibody-associated disorders, represents a distinctive subset within the spectrum of inflammatory demyelinating disorders. This category is distinct from both multiple sclerosis (MS) and neuromyelitis optica spectrum disorders (NMOSD) due to its peculiar pathophysiological mechanisms [1]. The core pathophysiological mechanism of MOGAD is the aberrant production of autoantibodies targeting the MOG, a key component of the myelin sheath surrounding nerve fibres. This immune response triggers a cascade of events, resulting in immune-mediated damage to oligodendrocytes, the cells responsible for producing myelin, ultimately manifesting in demyelination. This disruption in myelin integrity explains the characteristic clinical manifestations observed in these patients.

MOGAD signifies a unique subset of inflammatory demyelinating disorders, distinct from MS and NMOSD. The pathogenesis of MOGAD involves the production of autoantibodies targeting MOG, which leads to immune-mediated damage to oligodendrocytes resulting in demyelination [2].

Despite its relatively recent description, MOGAD has rapidly gained attention within the medical community owing to its heterogeneous clinical presentation and variable disease outcomes. The clinical spectrum of the disease spans a wide array of neurological manifestations, ranging from isolated optic neuritis and transverse myelitis to more complex syndromes such as acute disseminated encephalomyelitis (ADEM) and encephalitis. Furthermore, the prognosis of MOGAD exhibits considerable variation, with some patients experiencing monophasic disease courses, while others observing relapsing-remitting or even progressive disease patterns. This clinical diversity emphasises the interplay between immune dysregulation and neuroinflammation in MOGAD pathogenesis, necessitating further research to understand the underlying mechanisms driving this disease heterogeneity.

## Case presentation

A 21-year-old female, with no known comorbidities, presented with bilateral painful decreased vision three years prior to admission. The vision loss was insidious in onset, progressive, and severe, with no history of ocular or head trauma. Subsequent evaluation at an external centre specialising in ophthalmology revealed bilateral optic atrophy and compromised posterior circulation on magnetic resonance imaging (MRI) of the brain and optic nerves. However, the patient's medical records from the external centre were unfortunately unavailable for review.

**Figure 1:**
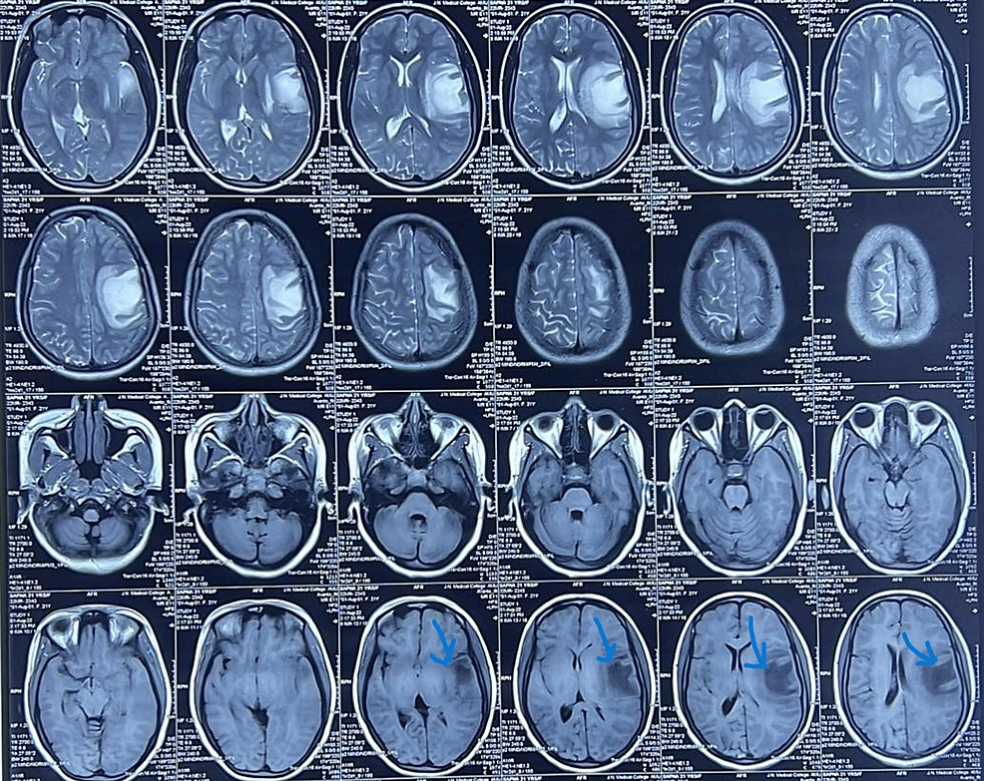
A large altered signal intensity area involving the left fronto-parieto-temporal lobe appearing hypodense on T1/T2/FLAIR with incomplete peripheral restriction on DWI. DWI: Diffusion-weighted imaging; FLAIR: Fluid‐attenuated inversion recovery

The patient was subsequently admitted to our centre with chief complaints of weakness and numbness in her bilateral lower limbs, along with urinary retention. On neurological examination, we found bilateral lower limb weakness (3/5 power) and intact upper limb strength. Magnetic resonance imaging (MRI) of the brain and cervical spine subsequently demonstrated a large altered signal intensity area involving the left fronto-parieto-temporal lobe appearing hypodense on T1/T2/fluid-attenuated inversion recovery (FLAIR) with incomplete peripheral restriction on diffusion-weighted imaging (DWI) and incomplete ring enhancement post contrast with perilesional edema (Figure 1). Mildly bulky left optic nerve at the level of chiasma and edema involving the cord extending from the level of T4-T8 vertebrae were also noted. The acute demyelinating encephalomyelitis on chronic bilateral optic neuritis and extensive spinal cord involvement raise suspicion for NMOSD or MOGAD.

Subsequent evaluations revealed positive anti-MOG antibodies in the absence of anti-aquaporin-4 (AQP4) antibodies, thus confirming the diagnosis of MOGAD. Treatment was initiated with pulse steroid therapy, followed by oral methylprednisolone and antiepileptic medication for seizure prophylaxis. Long-term management included oral immunomodulator therapy with azathioprine, leading to significant clinical improvement and neurological recovery during follow-up visits.

## Discussion

MOGAD presents distinctive challenges in diagnosis and management due to its unpredictable clinical manifestations and overlapping features with other demyelinating disorders [3]. Optic neuritis, transverse myelitis, and acute demyelinating encephalomyelitis are common clinical phenotypes observed in MOGAD, compelling careful clinical and radiological evaluation to distinguish it from MS and NMOSD [4].

Serological testing for anti-MOG antibodies has emerged as a beneficial tool in the diagnosis of MOGAD [5], with positive results providing supportive evidence for the disease. However, it must be noted that negative antibody testing does not exclude the diagnosis. This emphasises the importance of integrating clinical, radiological, and serological findings in the diagnostic protocol.

Immunosuppressive therapy with corticosteroids, intravenous immunoglobulins (IVIG), and immunomodulators such as azathioprine and mycophenolate mofetil (MMF) forms the cornerstone of treatment for MOGAD. Early initiation of immunosuppressive therapy is vital for delaying disease progression and reducing the risk of relapses [6].

Long-term management of MOGAD involves close monitoring of disease activity, neurological function, and treatment response. Regular follow-up visits, including clinical assessments, serological testing, and neuroimaging, are essential for boosting therapeutic strategies and limiting disease-related morbidity [7].

In addition to the challenges in diagnosis and management highlighted above, it is important to acknowledge the heterogeneity of MOGAD presentations. The clinical manifestations can vary widely among individuals, both in terms of severity and specific neurological symptoms experienced. This variability underscores the need for personalized approaches to diagnosis and treatment, as what works for one patient may not be as effective for another.

Furthermore, the evolving understanding of the pathophysiology of MOGAD adds to the complexity of its management. While anti-MOG antibodies have been recognised as a key biomarker, the definite mechanisms by which they contribute to disease development and progression are still uncertain. This ongoing research is essential for refining diagnostic criteria, identifying novel therapeutic targets, and ultimately improving outcomes for patients.

The impact of MOGAD extends beyond the neurological symptoms themselves, affecting various lifestyle aspects. For most patients, the ambiguity surrounding their condition and the potential for relapses can lead to significant psychological distress. Providing comprehensive support that addresses both the physical and mental health aspects of care is essential for optimizing patient well-being.

Additionally, the management of MOGAD requires multidisciplinary collaboration among healthcare professionals, including neurologists, psychologists, physiotherapy specialists, and ophthalmologists. This interdisciplinary approach ensures that patients receive comprehensive care that caters to the diverse range of symptoms associated with the condition.

Finally, ongoing research efforts aimed at further understanding MOGAD and refining treatment strategies are essential for promoting advanced clinical care. This includes developing new therapeutic agents, refining preexisting treatment protocols, and exploring potential biomarkers that can aid in early diagnosis and prognostication [8].

## Conclusions

In conclusion, the importance of recognizing and managing MOGAD requires a thorough multidisciplinary approach. Clinical suspicion and comprehensive diagnostic workup are paramount in successful management. Continued research efforts and collective initiatives are warranted to further elucidate the pathogenesis, improve diagnostic algorithms, and optimize therapeutic strategies for MOGAD, ultimately improving prognostic outcomes.
